# The Stability of G6PD Is Affected by Mutations with Different Clinical Phenotypes

**DOI:** 10.3390/ijms151121179

**Published:** 2014-11-17

**Authors:** Saúl Gómez-Manzo, Jessica Terrón-Hernández, Ignacio De la Mora-De la Mora, Abigail González-Valdez, Jaime Marcial-Quino, Itzhel García-Torres, America Vanoye-Carlo, Gabriel López-Velázquez, Gloria Hernández-Alcántara, Jesús Oria-Hernández, Horacio Reyes-Vivas, Sergio Enríquez-Flores

**Affiliations:** 1 Laboratorio de Bioquímica Genética, Instituto Nacional de Pediatría, México D.F. 04530, Mexico; E-Mails: yssterron@hotmail.com (J.T.-H.); ignaciodelamora@yahoo.com.mx (I.D.M.-D.M.); itzheltorres@hotmail.com (I.G.-T.); america_vc@yahoo.com.mx (A.V.-C.); glv_1999@yahoo.com (G.L.-V.); joria@bq.unam.mx (J.O.-H.); hreyesvivas@yahoo.com.mx (H.R.-V.); 2 Departamento de Biología Molecular y Biotecnología, Instituto de Investigaciones Biomédicas, Universidad Nacional Autónoma de México, México D.F. 04510, Mexico; E-Mail: abigaila@correo.biomedicas.unam.mx; 3 Cátedras CONACyT, Instituto Nacional de Pediatría, México D.F. 04530, Mexico; E-Mail: jmarcialqu@conacyt.mx; 4 Departamento de Bioquímica, Facultad de Medicina, Universidad Nacional Autónoma de México, México D.F. 04510, Mexico; E-Mail: galcantara72@yahoo.com.mx

**Keywords:** glucose-6-phosphate dehydrogenase (G6PD) deficiency, variants, recombinant G6PD enzymes, G6PD-deficient *E. coli*, protein stability, thermostability, structural characterization, structural NADP^+^

## Abstract

Glucose-6-phosphate dehydrogenase (G6PD) deficiency is the most common enzyme deficiency worldwide, causing a wide spectrum of conditions with severity classified from the mildest (Class IV) to the most severe (Class I). To correlate mutation sites in the G6PD with the resulting phenotypes, we studied four naturally occurring G6PD variants: Yucatan, Nashville, Valladolid and Mexico City. For this purpose, we developed a successful over-expression method that constitutes an easier and more precise method for obtaining and characterizing these enzymes. The *k*_cat_ (catalytic constant) of all the studied variants was lower than in the wild-type. The structural rigidity might be the cause and the most evident consequence of the mutations is their impact on protein stability and folding, as can be observed from the protein yield, the *T*_50_ (temperature where 50% of its original activity is retained) values, and differences on hydrophobic regions. The mutations corresponding to more severe phenotypes are related to the structural NADP^+^ region. This was clearly observed for the Classes III and II variants, which became more thermostable with increasing NADP^+^, whereas the Class I variants remained thermolabile. The mutations produce repulsive electric charges that, in the case of the Yucatan variant, promote increased disorder of the *C*-terminus and consequently affect the binding of NADP^+^, leading to enzyme instability.

## 1. Introduction

Glucose-6-phosphate dehydrogenase (G6PD, EC 1.1.1.49) catalyzes the oxidation of glucose-6-phosphate to 6-phosphogluconolactone concomitantly with the reduction of NADP^+^ to NADPH in the pentose phosphate pathway, which represents the only source of NADPH in erythrocytes. G6PD regulates the level of reduced glutathione in the cell by producing NADPH [1,2], which is crucial for protecting erythrocytes against oxidative damage. G6PD deficiency is the most frequent enzymopathy in humans and affects more than 400 million people in the world [3], causing a wide spectrum of clinical manifestations, from asymptomatic individuals to cases with neonatal jaundice, acute hemolytic episodes and chronic non-spherocytic hemolytic anemia, among other problems [4].

G6PD deficiency is genetically heterogeneous, with nearly 160 mutations reported. Most are point mutations leading to single amino acid substitutions, producing G6PD variants [5]. G6PD variants are usually classified according to their residual enzymatic activity and the hematological parameters of the patients, ranging from Class I (the most severe, with less than 5% residual activity) to Class IV (the mildest form). Interestingly, more than half the known mutations that produce Class I variants are clustered in exon 10, which encodes amino acid residues localized near the dimer interface [3,6,7] and close to the structural NADP^+^ binding site. The latter region is considered important for the stability and integrity of the functional enzyme [8].

To understand the detrimental effects of mutations on the functional and structural properties of human G6PD, in this study, we characterized four G6PD variants found in the Mexican population representing Class I to III. We included two naturally occurring Class I variants, G6PD Yucatan (nt 1285 A→G, K429E) and G6PD Nashville (nt 1178 G→A, R393H), both with mutations located in exon 10 [9,10,11]. The G6PD Valladolid variant (nt 406 C→T, R136C), the second most frequent haplotype found in Mexican Mestizos, is a non-polymorphic Class II variant [12]. Finally, the G6PD Mexico City variant (nt 680 G→A, R227Q) has only been found in Mexico corresponding to Class III [13].

To explore the underlying characteristics at the molecular level of the Yucatan, Nashville, Valladolid and Mexico City G6PD variants and their relationships with clinical manifestations, we constructed and expressed recombinant G6PD enzymes using the G6PD-deficient *E. coli* BL21 bacterial strain and the pET-3a plasmid. Wild-type (WT) G6PD and the four mutant enzymes were expressed under a variety of conditions and purified to homogeneity to perform detailed studies on their biochemical and structural properties.

In this work, we report the functional and structural parameters of three G6PD variants that have never been characterized before and compare them with the corresponding values of the WT human enzyme. Additionally, we have re-characterized the G6PD Nashville variant, which was previously reported by Wang *et al*. [6,14]. This latter variant was included to compare it with the G6PD Yucatan variant because both are classified as Class I. Moreover, we developed a successful over-expression system based on the use of *E. coli* strain BL21 and the pET-3a plasmid. This system allows easier and more precise characterization of the recombinant enzymes because the bacterium lacks endogenous G6PD enzyme. Our results show that the location of the mutation affects the catalytic properties, stability and structure of G6PD and that these changes are closely associated with the clinical presentation of its deficiency.

## 2. Results

### 2.1. Construction of E. coli BL21(DE3)Δzwf::kan^r^ Mutant

The *E. coli* BL21(DE3)Δ*zwf*::kan^r^ deletion mutant was generated by genetic recombination mediated by P1*vir* transduction [15]. One clone of *E. coli* K-12 from the Keio collection of in-frame single-gene knockout mutants contains a *zwf* gene (1446 bp) deletion (*zwf* is the *E. coli* G6PD gene) replaced by the kanamycin resistance gene (1326 bp) [16]. This clone was used as donor cells, and the *E. coli* BL21(DE3) strain was used as the acceptor cells. Two clones were selected and named *E. coli* BL21(DE3)Δ*zwf*::kan^r^.

**Figure 1:**
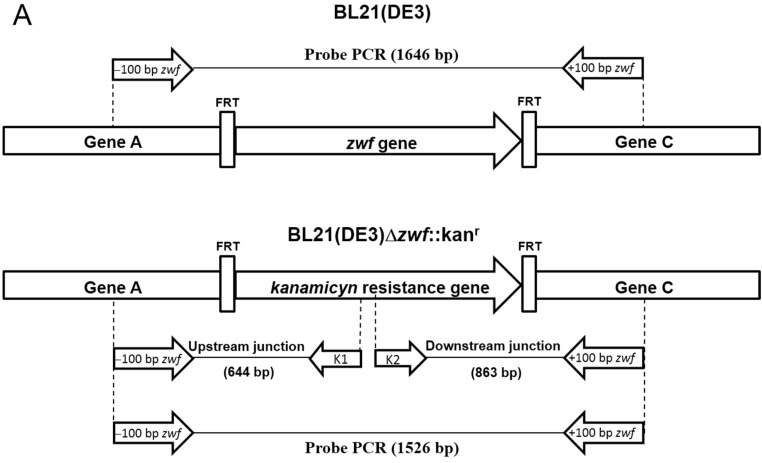

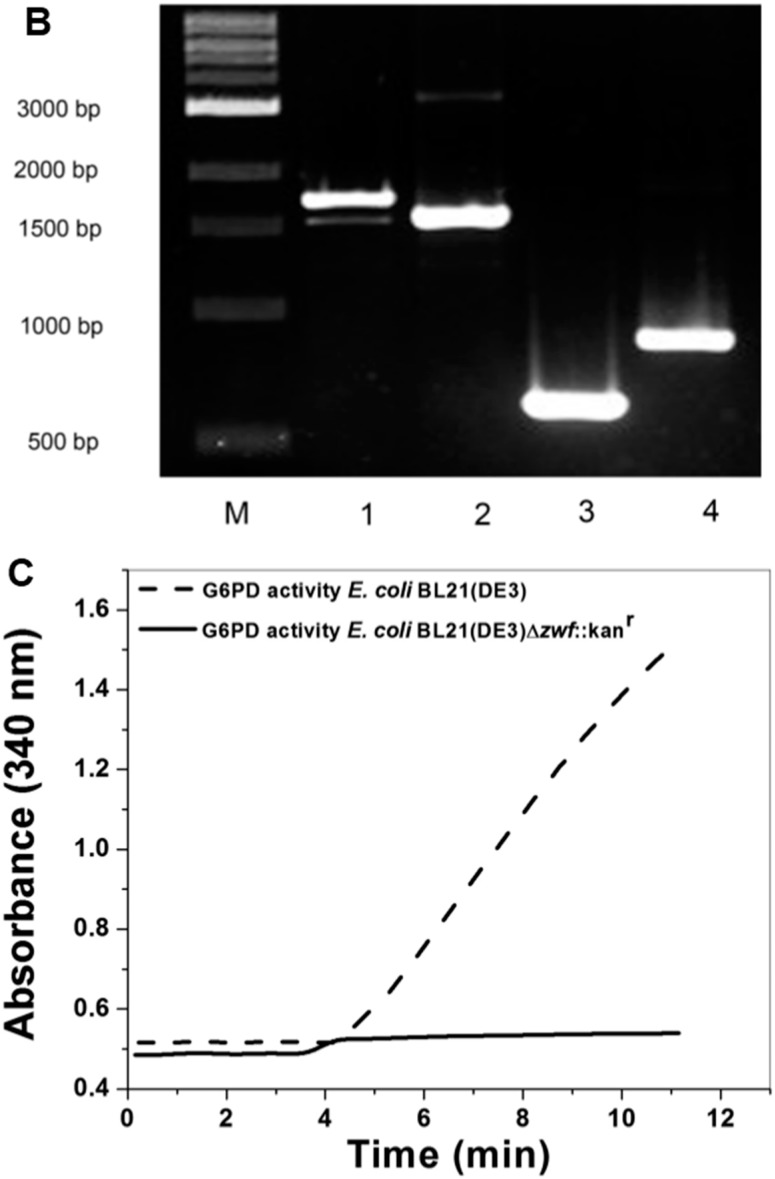
Construction of *E. coli* BL21(DE3)Δ*zwf*::kan^r^ mutant. (**A**) Representation of gene arrangement in *E*. *coli* BL21(DE3)Δ*zwf*::kan^r^ generated by homologous recombination. The primers used for mutant analysis and the PCR probe amplified for each construct are shown. The specific primers were located upstream and downstream from the *zwf* gene (−100 bp *zwf* and +100 bp *zwf*), and the primers for kanamycin resistance gene amplification (K1 and K2) were internal; (**B**) Generation of *E. coli* BL21(DE3)Δ*zwf*::kan^r^ by knockout of the *zwf* gene was confirmed by PCR. **M**: Molecular weight marker; Lane **1** shows the PCR product using gDNA from *E. coli* BL21(DE3) as a template and the primers −100 bp *zwf* Forward (Fw) and +100 bp *zwf* Reverse (Rv); Lane **2** shows the PCR product with gDNA from *E. coli* BL21(DE3)Δ*zwf*::kan^r^ and the primers −100 bp *zwf* Fw and +100 bp *zwf* Rv; Lane **3** corresponds to the PCR product using gDNA from *E. coli* BL21(DE3)Δ*zwf*::kan^r^ and the specific primers +100 bp *zwf* Rv and K1; Lane **4** corresponds to the PCR product using gDNA from *E. coli* BL21(DE3)Δ*zwf*::kan^r^ and the primers K2 and +100 bp *zwf* Rv. In all cases, 100 ng of gDNA was used as template; and (**C**) Specific activity of glucose-6-phosphate dehydrogenase (G6PD) measured in crude extract of both *E. coli* BL21(DE3) and *E. coli* BL21(DE3)Δ*zwf*::kan^r^ cells. The reaction was started with 10 µL of crude extract in the mixture reaction containing 1 mM G6P and 2 mM NADP^+^ in 50 mM Tris-HCl buffer, pH 8.0.

To confirm that the *zwf* gene was knocked out in *E. coli* BL21(DE3), we employed two different strategies. In the first strategy, PCR assays were performed using the specific primers −100 bp *zwf* Forward (Fw) and +100 bp *zwf* Reverse (Rv) (Supplementary Table S1 and Figure 1A) with either *E. coli* BL21(DE3) or *E. coli* BL21(DE3)Δ*zwf*::kan^r^ genomic DNA (gDNA) as the template. In the case of *E. coli* BL21(DE3), a PCR product of approximately 1646 bp was obtained (Figure 1B, Lane 1), which is consistent with the expected 1446 bp fragment of the *zwf* gene from *E. coli* BL21(DE3). When gDNA from *E. coli* BL21(DE3)Δ*zwf*::kan^r^ was used as the template, a PCR product of 1526 bp was obtained (Figure 1B, Lane 2), which is consistent with the kanamycin resistance gene (1326 bp), and which was verified by sequencing (Figure 1A). Furthermore, two PCR reactions were performed using internal K1 and K2 primers for the *kanamycin* gene (Figure 1A). In both cases, we obtained PCR products (Figure 1B, Lanes 3 and 4) corresponding to the expected fragments, and therefore we can confirm that the *zwf* gene from *E. coli* BL21(DE3) was knocked out by genetic recombination. In the second strategy, to verify the genetic recombination forming the *E. coli* BL21(DE3)Δ*zwf*::kan^r^ deletion mutant, we grew both *E. coli* BL21(DE3) and *E. coli* BL21(DE3)Δ*zwf*::kan^r^. The cells were lysed, and the crude extract was used to measure the specific activity of G6PD. The results demonstrated that *E. coli* BL21(DE3) cells containing the *zwf* gene showed specific G6PD activity of 1.6 IU·mg^−1^, whereas the BL21(DE3)Δ*zwf*::kan^r^ showed no G6PD activity (Figure 1C). Based on these results, we obtained *E. coli* BL21(DE3)Δ*zwf*::kan^r^ cells with *g6pd* knocked out, which allowed us to perform expression assays for the remainder of the studies in this work.

### 2.2. Construction, Expression and Purification of Recombinant Human G6PD

**Figure 2:**
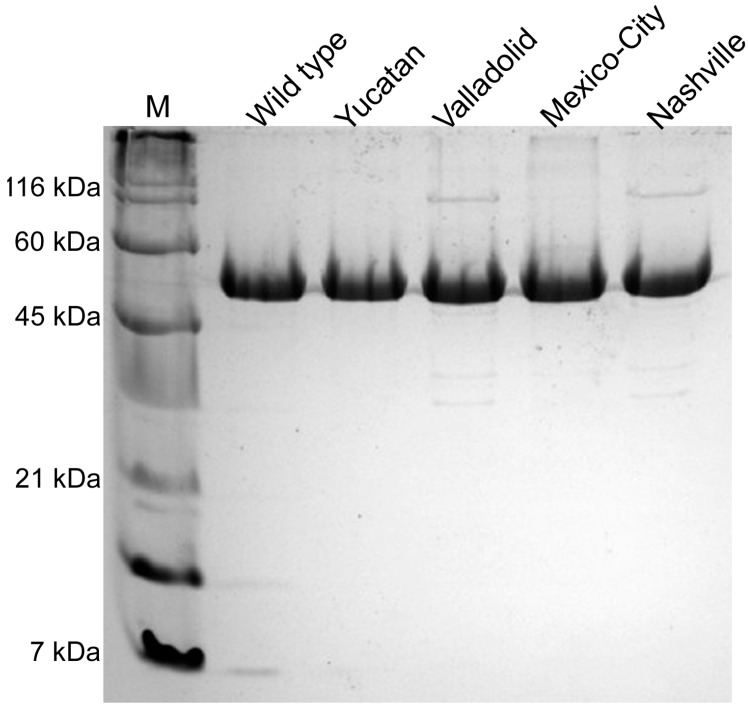
Purification of human recombinant glucose-6-phosphate dehydrogenase (G6PD) enzymes. SDS-PAGE of purified G6PD enzymes. **M**: molecular weight marker; Lanes **2**–**5**: WT G6PD and G6PD variants. Each lane was loaded with 10 µg of protein and visualized using coomassie brilliant blue.

Plasmid with a sequence that codifies for a (His)_6_-tag and a tobacco etch virus protease recognition sequence introduced at the amino-terminus of the protein (pET-HisTEVP-*g6pd*) [17], was used as the template for site-directed mutagenesis. Vectors constructed with the desired mutations in WT G6PD were used to transform competent *E. coli* BL21(DE3)Δ*zwf*::kan^r^ cells. To find the best overexpression conditions, we tested different concentrations of isopropyl-β-d-thiogalactopyranoside (IPTG) and growth temperatures (see Materials and Methods Section). The expression of the G6PD enzymes was time-dependent, with maximal specific activity at 18 h in most cases, an exception being the NashvilleG6PD variant, with an optimal expression time of 6 h. A specific activity of 1.6 IU·mg^−1^ was obtained for the crude extract with WT G6PD grown in 0.5 mM IPTG at 25 °C and 18 h of expression time. We obtained specific activities of 1.10, 0.77, 0.50 and 0.09 IU·mg^−1^ for the Yucatan, Valladolid, Mexico City and Nashville G6PD mutants, respectively. However, the specific activity in the crude extract of the Nashville G6PD was different from previously reported data [6]. The WT G6PD enzyme showed the highest specific activity (1.60 IU·mg^−1^) compared to all the mutants used in this study (Supplementary Figure S1). The specific activities of the Yucatan, Valladolid, Mexico City and Nashville G6PDs were 70%, 50%, 50% and 5% of the WT G6PD activity, respectively. G6PD recombinants were purified, and a single band with greater than 95% purity was obtained for each mutant (Figure 2) as evaluated by SDS-PAGE. For the Nashville G6PD variant, we obtained 90% purity because this enzyme was purified in one step using an affinity column due to its loss of activity during purification, specifically during Q-Sepharose ion-exchange chromatography.

All the mutants were purified following identical purification procedures, and the specific activities of the purified enzymes are shown in Table 1. We obtained approximately 5 mg of pure protein from 2 liters of *E. coli* BL21(DE3)Δ*zwf*::kan^r^ culture for the majority of the recombinants, except for the Nashville G6PD, where the yield was approximately 1.7 mg of pure protein. The yield from the purification of the Mexico City G6PD (Class III) was approximately 57%, which is very similar to the yield obtained for the WT G6PD. The Yucatan and Valladolid G6PD variants gave yields of approximately 30%, and the Nashville variant gave the lowest yield (13%). As a general effect of the mutations, we noted that, independently of their location in the enzyme, each mutation induced a change in the stability of the protein. This effect is evident in the reduced specific activity of all the mutants compared to WT G6PD.

**Table 1:** Summary of the purification of recombinant human G6PD enzymes.

G6PD	Total Protein (mg)	Specific Activity (IU·mg^−1^)	Total Activity (IU)	Yield (%)
WT	4.8	224	1075	61
Yucatan (I)	4.2	132	554	35
Valladolid (II)	4.3	92	395	29
Mexico City (III)	4.4	175	770	57
* Nashville (I)	1.7	103	175	13

Values are the results for a typical expression purification experiment; the results vary <10% from batch to batch. G6PD activity was measured under the standard conditions described in Materials and Methods. Each value was obtained from the corresponding purification summary from 2 L of culture. The numbers in parentheses indicate the class of each variant. * Indicates the re-characterized Nashville G6PD previously purified by Wang *et al.* [18].

### 2.3. Determination of Steady-State Kinetic Parameters

Kinetic analyses were performed on the four clinical variants and WT G6PD. WT G6PD showed the highest *k*_cat_ (catalytic constant) value (233 s^−1^) of all the enzymes included in this study. The *k*_cat_ values obtained for the G6PD variants were as follows: Yucatan (138 s^−1^), Valladolid (96 s^−1^), Mexico City (182 s^−1^) and Nashville (119 s^−1^) (Table 2). These data indicate that catalytic efficiency is affected differentially by these mutations. For example, the Mexico City G6PD (Class III variant) was the least affected enzyme, with a *k*_cat_ value 1.3-fold lower than that of the WT G6PD. By contrast, the Nashville and Valladolid G6PDs (Classes I and II variants, respectively) were the most affected enzymes with respect to their *k*_cat_ values, which were 1.95- and 2.42-fold lower than the *k*_cat_ value of the control enzyme. The *K*_mG6P_ (Michaelis-Menten constant for G6P substrate) value of the Yucatan G6PD (Class I) was very close to that of the control enzyme. It is especially striking that the Yucatan mutant shows parameters almost identical to the WT enzyme, considering the clinical severity of this Class I variant. The Valladolid and Mexico City G6PD variants show more affinity for substrate, with *K*_mG6P_ values of 21.5 and 24.9 µM, respectively. However, the Nashville G6PD variant was the only mutant that lost more than two-fold affinity for the G6P substrate (90.6 µM) with respect to the WT G6PD enzyme. In contrast, the *K*_m_ values obtained for the catalytic coenzyme (NADP^+^) were very similar in most cases (approximately 6 to 9 µM), an exception being the G6PD Nashville variant (31.2 µM), which showed approximately five-fold lower affinity compared with the WT G6PD enzyme.

**Table 2:** Steady-state kinetic parameters of human recombinant G6PD proteins.

G6PD	*k*_cat_ (s^−1^)	*K*_mG6P_ (µM)	*K*_mNADP_^+^ (µM)	*k*_cat_/*K*_mG6P_ (s^−1^·M^−1^)	*k*_cat_/*K*_mNADP_*^+^* (s^−1^·M^−1^)
Wild-type	233	38.5	6.2	6.0 × 10^6^	37.8 × 10^6^
Yucatan (I)	138	39.9	6.4	3.5 × 10^6^	21.7 × 10^6^
Valladolid (II)	96	21.5	3.6	4.4 × 10^6^	26.2 × 10^6^
Mexico City (III)	182	24.9	9.1	7.3 × 10^6^	19.1 × 10^6^
Nashville (I)	119	90.6	31.2	1.3 × 10^6^	3.8 × 10^6^

The values for the activity of the G6PD enzymes are averages of three independent experiments; in all cases, the standard errors were ±5%. The numbers in parentheses indicate the class of each variant.

### 2.4. Structural Characterization of G6PD Enzymes

**Figure 3:**
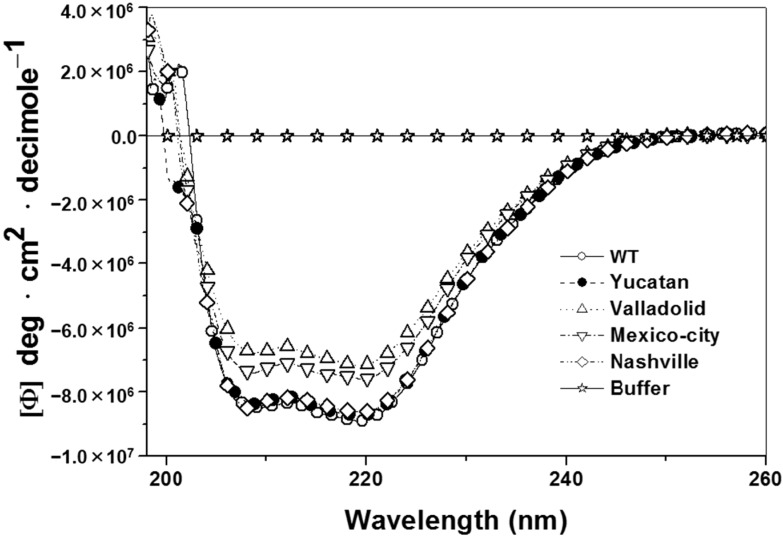
Structural characterization of human recombinant wild-type (WT) G6PD and mutants. Far-UV circular dichroism (CD) spectra of WT G6PD and mutants are shown. The protein concentration was 0.8 mg/mL in all cases. The experiments were performed in duplicate; standard errors were less than 5%.

Because the G6PD variant enzymes have diminished catalytic efficiency, it was necessary to determine whether these conformational changes are the result of a wider structural disruption or a local effect. The secondary structures of the WT G6PD and the clinical variants were evaluated spectroscopically by circular dichroism (CD). As shown in Figure 3, the CD spectra of the mutant enzymes were comparable in pattern and intensity to the WT enzyme, indicating that the mutations did not significantly alter the secondary structure of the proteins.

### 2.5. Analysis of the Stability of the G6PD Enzymes

**Figure 4:**
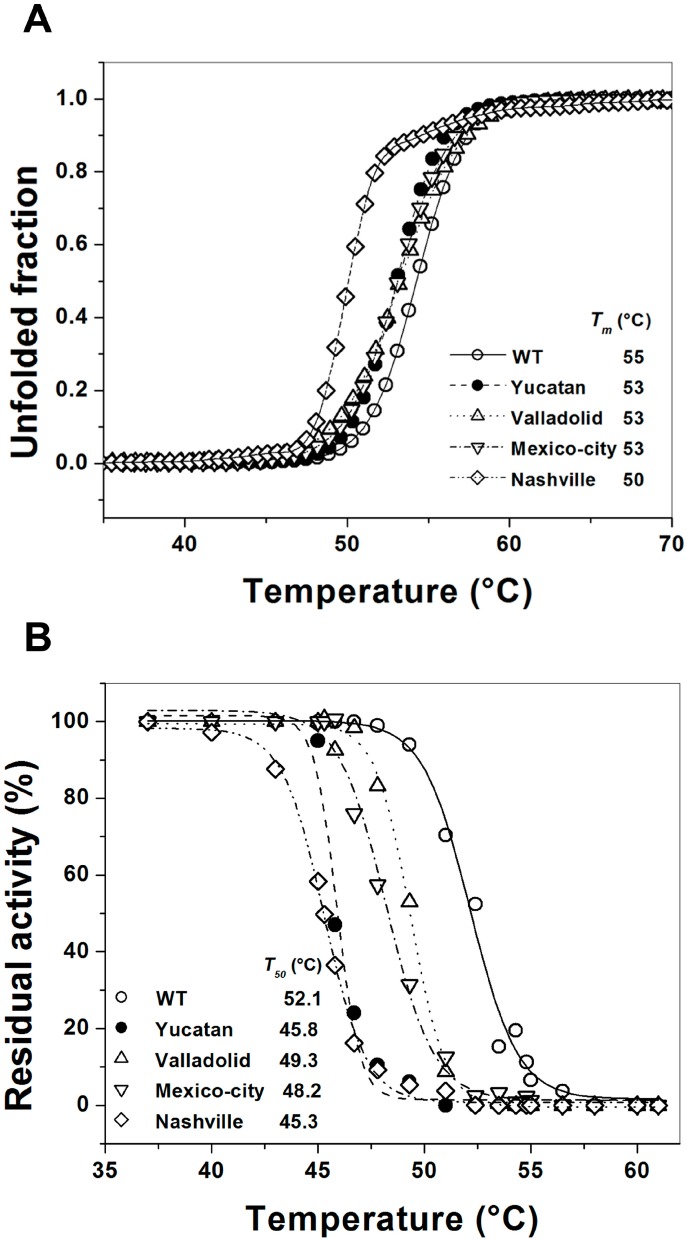
Thermal stability of human G6PD enzymes. (**A**) Thermal unfolding of WT G6PD and the mutants (0.8 mg/mL) in 25 mM NaPO_4_ pH 7.4 was monitored by recording the change in CD signal at 222 nm at different temperatures ranging from 20 to 90 °C. The unfolded fraction of protein and the *T*_m_ (melting temperature midpoint of the transition values) (inset) were calculated as previously reported [22]; and (**B**) Thermal inactivation assays of WT G6PD and the mutants after incubation for 20 min at the indicated temperature. The *T*_50_ (temperature where 50% of its original activity is retained) after incubation at different temperatures for 20 min is shown. In all cases, 200 ng of total protein was used. The assays were performed in duplicate; standard errors were lower than 5%.

Protein stability has been extensively studied in human G6PD, and it has already been demonstrated that several mutations can induce G6PD deficiency by decreasing its stability [7,18,19,20]. To assess whether the diminished catalytic activity of the studied mutant enzymes was due to an alteration in protein stability, we evaluated the thermal denaturation of the four G6PD enzymes. The global stability of the proteins was followed as the change in the CD signal at 222 nm. The results indicate a two-state process with a *T*_m_ (melting temperature midpoint of the transition) of 54.3 °C for the WT G6PD enzyme (Figure 4A). This result differs marginally from the recently published *T*_m_ value of 51.5 °C obtained for recombinant G6PD-HisTEVP [17]. However, it is consistent with a previously reported *T*_m_ value for the WT G6PD enzyme of approximately 55 °C [14]. The *T*_m_ was 54.3 °C for WT G6PD and 53 °C for the Yucatan, Valladolid and Mexico City G6PDs. This change in the *T*_m_ was not significant; however, the structural stability of the Nashville G6PD enzyme shows a strong difference because the *T*_m_ was 50 °C, 5 °C below the value obtained for the WT G6PD enzyme, indicating that this enzyme is more sensitive to temperature denaturation.

However, due to the lack of reports about heat inactivation profiles for the Yucatan, Valladolid, and Mexico City G6PDs and because the only profile for the Nashville G6PD was for semi-purified enzyme [6], thermal inactivation assays were used to evaluate the stability of the active site of the G6PD enzymes. The WT G6PD enzyme displayed a *T*_50_ of 55 °C. The *T*_50_ values obtained without NADP^+^ were 52 °C for WT and 45.8, 49.3, 48.2 and 45.3 °C for the Yucatan, Valladolid, Mexico City and Nashville G6PD variants, respectively. Then, we determined the *T*_50_ values with 5 µM NADP^+^ (Figure 4B), as shown the Valladolid and Mexico City G6PD mutants were the most heat-resistant, with a *T*_50_ of approximately 49 °C, 3 °C below the value obtained for WT G6PD. In contrast, the Yucatan and Nashville G6PD variants were the most thermolabile proteins, with a *T*_50_ of 45 °C for both mutants, a shift of 7 °C with respect to the WT G6PD enzyme.

**Figure 5:**
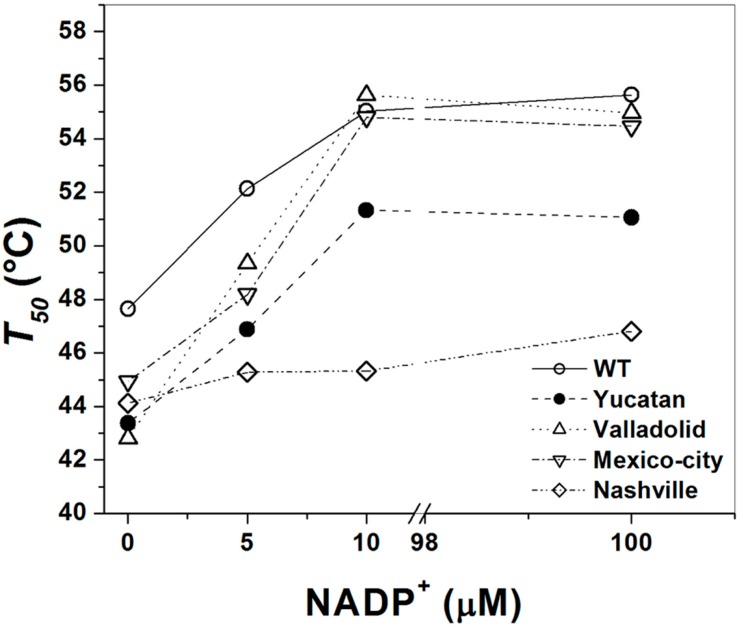
Effect of NADP^+^ on the thermoinactivation of recombinant G6PD enzymes. *T*_50_ is plotted against the NADP^+^ concentration. The WT and mutant enzymes were incubated at different NADP^+^ concentrations. In all cases, 200 ng of total protein was used.

Furthermore, the thermostability of the enzymatic activity of pure WT G6PD and the four variants was examined in the presence of different concentrations of NADP^+^ (1–500 µM), which has been widely used as a stabilizer for human G6PD enzymes [8,18]. The effect of NADP^+^ on thermostability (Figure 5) shows again that the WT G6PD enzyme is more stable than the variants, with approximately 3 and 5 °C of difference in the *T*_50_ NADP^+^-dependent stabilization was observed in the WT, Valladolid and Mexico City G6PD enzymes. However, in the Yucatan and Nashville G6PD variants (Class I), no protective effect was observed when NADP^+^ was increased. The *T*_50_ values increased up to 10 °C with 500 µM NADP^+^ for the WT, Valladolid and Mexico City G6PD enzymes. However, at all concentrations of NADP^+^, the Yucatan and Nashville G6PD variants were less stable than WT. We observed that increased NADP^+^ concentration had no ability to stabilize either of the Class I mutant enzymes, indicating that these mutants lost their affinity for structural NADP^+^ and suggesting that these enzymes have become structurally unstable and more relaxed.

### 2.6. Structural Characterization of the G6PD Enzymes

**Figure 6:**
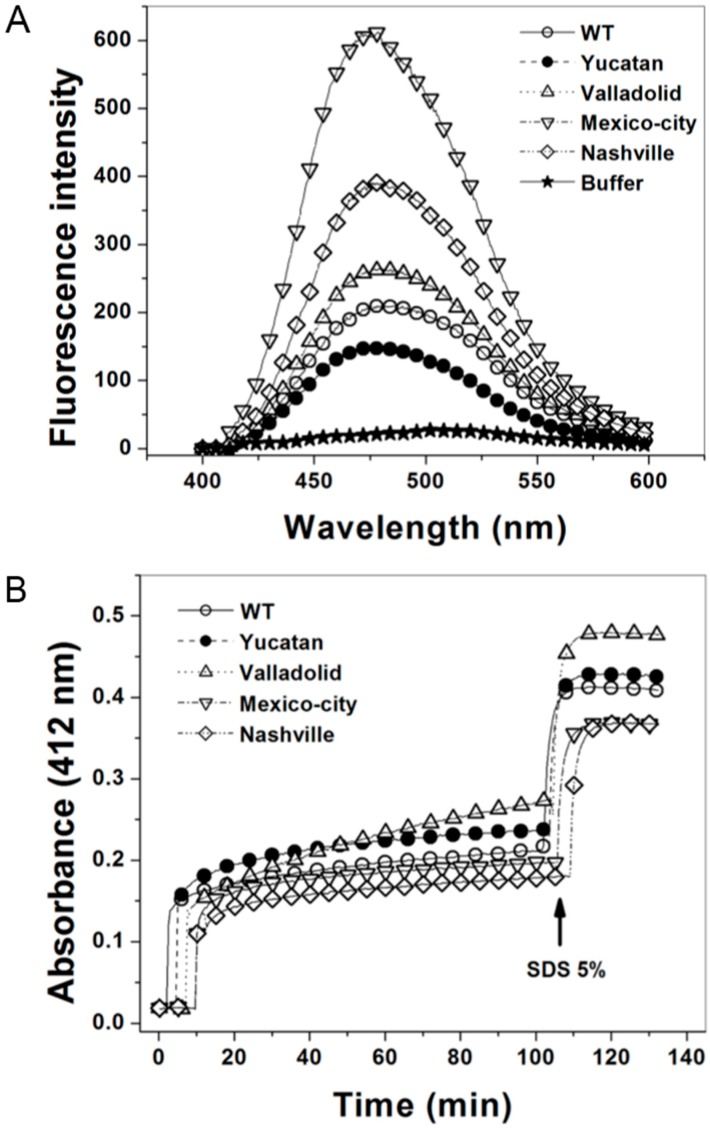
8-Anilinonaphthalene-1-sulphonate (ANS) and 5,5'-dithiobis-2-nitrobenzoic acid (DTNB) accessibility assays of WT G6PD and the four clinical variants. (**A**) ANS fluorescence spectra of WT G6PD and the four clinical mutants were obtained using an excitation wavelength of 395 nm and recording emission spectra from 400 to 600 nm; the final concentrations of ANS and enzyme were 165 μM and 400 μg/mL, respectively. Values obtained from buffer containing ANS without protein (open stars) were subtracted from the recordings with protein; and (**B**) The derivatization of Cys residues in G6PD (200 µg/mL) in 1 mM DTNB was followed spectrophotometrically at 412 nm. The arrow indicates the time of addition of 5% SDS, after which the number of Cys per monomer was calculated for the enzymes. Each trace is the average of two independent experiments.

To confirm that the G6PD variants contain structural alterations, we analyzed their capacity to bind 8-anilinonaphthalene-1-sulphonate (ANS) [21]. The fluorescence of ANS was four-fold higher in the Mexico City G6PD mutant than in the WT G6PD. Furthermore, the Nashville and Valladolid G6PD variants were 2.6- and 1.8-fold higher than the WT G6PD enzyme, respectively. This increased fluorescence indicates that rearrangements of the hydrophobic pockets exist in the mutant enzymes, which have moved to a more solvent-exposed environment (Figure 6A). It is important to note that none of the mutants showed a tendency to aggregate. Based on these results, we conclude that the exposure of hydrophobic pockets in the mutant enzymes is the result of a more relaxed 3D structure.

We also evaluated the structural compactness of the WT G6PD and the mutants by following their Cysteine (Cys) accessibility using 5,5'-dithiobis-2-nitrobenzoic acid (DTNB) [23]. Time-courses of the chemical modification of G6PD enzyme showed that WT G6PD and all the mutants showed rapid derivatization of 2.3 Cys per monomer in the presence of DTNB (Figure 6B). After this initial phase, we observed a progressive increase in Cys derivatization, and after 120 min, we observed that the WT, Yucatan and Mexico City G6PD enzymes were slowly derivatized, with only 1.2, 1.5 and 1.0 Cys per monomer, respectively. However, for the Valladolid and Nashville G6PD mutants, 2.3 and 1.5 Cys per monomer were derivatized (Figure 6B). Finally, when all the enzymes were exposed to 5% SDS, rapid derivatization of 4.1, 4.2, 4.3, 3.7 and 4.1 Cys per monomer was observed for the WT, Yucatan, Valladolid, Mexico City, and Nashville G6PD enzymes, respectively. These results are consistent with the results observed in the G6PD crystallographic structure [8], where four Cys are not solvent accessible. This result suggests again that compared to the WT G6PD enzyme, the structural alterations caused by the mutations promote changes in the interactions of neighboring residues and force the misfolding of adjacent areas, which triggers accessibility of Cys to derivatization and opens space for the inclusion of ANS in hydrophobic patches that are exposed by the mutation.

## 3. Discussion

In this paper, we simplified the purification process of human G6PD by cloning the human *g6pd* gene and variants into a pET-HisTEVP over-expression vector. Protein expression was performed in a genetically modified *E. coli* BL21(DE3)Δ*zwf*::kan^r^ bacterial strain (Figure 1). This system allowed us to purify human recombinant G6PD, excluding endogenous G6PD activity from *E. coli* BL21(DE3), and to obtain sufficient quantities of purified protein to perform detailed biochemical and biophysical studies. Although all of the mutants were expressed under the best protein expression conditions, they exhibited lower specific activities than the WT enzyme (Supplementary Figure S1).

Mutations that cause severe phenotypes, such as chronic non-spherocytic hemolytic anemia, are classified as Class I variants and occur mainly in exons 10 and 11. These exons encode amino acids 380–430, located close to the dimer interface, which is stabilized by hydrophobic interactions, charge-charge interactions and salt bridges (*i.e.*, weak ionic bonds). All the variants caused by mutations located in this area show a striking reduction in thermal stability *in vitro* [3,6,7,24]. By contrast, the point mutations that cause Classes II and III variants are spread throughout the entire coding region. For example, G6PD Union (R454C) (Class II) is located in exon 11, and G6PD Mahidol (G163S), a Class III variant, is located in exon 6. Arginine 454 is a highly conserved solvent-exposed amino acid that is very important for maintaining G6PD structure and function [7]. Glycine163 is located at a tight turn between an α-helix and a β-sheet at the surface of the molecule, distant from the dimer interface and the binding sites for structural NADP^+^, substrate and coenzyme. The Valladolid G6PD (nt 406 C→T, R136C) is a Class II variant with the mutation located in exon 5, whereas the Mexico City G6PD (nt 680 G→A, R227Q) is a Class III variant with the mutation located in exon 7. We kinetically and biochemically characterized four variants, two severe Class I (Yucatan and Nashville), one Class II (Valladolid) and one Class III (Mexico City), to correlate their biochemical parameters with their stability and with the clinical manifestations observed in each variant.

An analysis of the yield obtained from the purification of each mutant (see Results Section 2.2 and Table 2) showed a relationship between the mutations and protein stability. The G6PD enzymes can thus be sorted by their protein stability from WT G6PD, Mexico City, Yucatan, Valladolid to Nashville (*i.e.*, from high stability to low stability). We noticed that all the mutations are located at different regions in the enzyme, all far away from the active site; however, all of them showed diminished specific activity. Therefore, it is probable that the instability of the mutants could be responsible for their diminished specific activity.

It is interesting to note that the kinetic parameters of WT G6PD enzyme were in close agreement with previously determined values [6,7,14,17,20,25,26,27]. The Classes II (G6PD Valladolid) and III (G6PD Mexico City) variants characterized in this work showed moderate changes in kinetic and structural parameters. All the kinetic parameters of the Valladolid G6PD are lower than the corresponding values of WT G6PD enzyme (*k*_cat_: 96 s^−1^ *vs.* *k*_cat_ of WT G6PD of 233 s^−1^), and the *K*_m_ values for both substrates (NADP^+^ and G6P) are also lower than for the WT enzyme. This decrease in *K*_m_ values has also been observed in the G6PD mutants Union (Class I) and Andalus (Class II) [20], and although this change appears favorable, it must be considered that even at saturation, these two mutant enzymes would show lower activity compared to the WT G6PD enzyme. The kinetic parameters found for the Mexico City G6PD variant are similar to the values previously reported for the Mahidol G6PD variant (Class III) [7]. However, the Mexico City G6PD variant was slightly less active (*k*_cat_ = 182 s^−1^) than WT G6PD (*k*_cat_ = 233 s^−1^) under saturating conditions. This mutant showed *K*_m_ values in the micromolar range for both substrates (G6P and NADP^+^). However, these values are lower than the *K*_m_ of WT G6PD.

There were no changes in the secondary structure of the Valladolid and Mexico City G6PDs in comparison with WT G6PD, as followed by CD spectra in the far UV region (Figure 3). Likewise, the unfolding patterns in response to thermal denaturation as measured by CD spectra were very similar between the Valladolid and Mexico City G6PDs and correspond to WT G6PD enzyme with a Δ*T*_m_ of 2 °C, suggesting that the observed point mutations have no drastic effect on the overall structural stability of the enzyme. However, thermal inactivation of the Valladolid and Mexico City G6PDs occurred at a lower temperature (by 5 °C) than thermal inactivation of WT G6PD enzyme (Figure 4B).

Furthermore, the thermostability of the Valladolid and Mexico City G6PD enzymes were examined with increasing temperature in the presence of varying concentrations of NADP^+^. NADP^+^-dependent stabilization was evident for the Valladolid and Mexico City G6PDs, as seen for WT G6PD, where the *T*_50_ values were higher by 10 °C (Figure 5). This protective effect is consistent with previous reports for WT G6PD, and A−, Union, Andalus and Wisconsin G6PD variants [6,8,18,20,28,29].

Finally, the fluorescence of the ANS assays showed that the Valladolid and Mexico City G6PD enzymes have more hydrophobic regions exposed to solvent than the WT G6PD enzyme (Figure 6). These changes detected in the thermal inactivation and fluorescence assays indicate that the Classes II and III mutant enzymes with low catalysis are less stable than WT G6PD, probably because the structural rigidity of the mutant enzymes is much lower.

Two naturally occurring Class I variants were included in this study, G6PD Yucatan (K429E) [9,10] and G6PD Nashville (R393H) [11], both with mutations in the same exon. The mutations K429E and R393H moderately affected the kinetic parameters of G6PDs Yucatan and Nashville. The *k*_cat_ values for the Yucatan and Nashville G6PDs were 138 and 119 s^−1^, respectively. However, the *K*_m_ values for G6PD Yucatan were very close to *K*_m_ of the WT G6PD enzyme. This similarity is especially striking in that this mutant exhibits almost identical parameters despite the clinical severity of the Yucatan G6PD variant. The same effect has been observed with the Plymouth G6PD variant (Class I) [7], which exhibits almost identical parameters despite the clinical severity of the Plymouth mutation. Nonetheless, the Nashville G6PD variant was the only mutant in this study that lost more than two-fold affinity for G6P as a substrate (Table 2) and five-fold affinity for the catalytic coenzyme NADP^+^ with respect to the WT G6PD enzyme. These changes in catalytic efficiency, especially in the Class I variants under saturating conditions, indicate that these mutants probably induce conformational changes that are transmitted to the active site and could be the predominant cause of the severe disease phenotype.

In order to assess whether those probable conformational changes are responsible for the loss of activity, we conducted a series of experiments to determine the secondary structure and global stability of WT G6PD and the pathological variants. Analysis of the secondary structure of the Class I variants showed no significant differences between the WT and the mutant enzymes (Figure 3). These data are similar to the results obtained for the Classes II and III variants. Similarly, stability assays of the Yucatan and Nashville G6PDs demonstrated that thermal denaturation occurred at similar temperatures similar to the WT G6PD enzyme. However, a higher degree of destabilization was observed with G6PD Nashville (Δ*T*_m_ of 5 °C), which is consistent with the kinetic parameters, showing that this variant is the most severely affected protein. Consistently with these observations, we determined thermal inactivation, and both the Yucatan and Nashville G6PD enzymes (Class I) were found to be significantly less stable than WT G6PD enzyme.

Furthermore, the thermostability of both Class I variants was examined with increasing temperature in the presence of varying concentrations of NADP^+^. NADP^+^-dependent stabilization was more evident in the Classes II and III variants and equal to the value for the WT G6PD. However, this protective effect was not observed in the Class I variants Yucatan and Nashville, and similar data have been reported for other Class I variants, such as G6PDs Wisconsin, Fukaya, Campinan Plymouth and Andalus [3,6,7]. This difference in the protective effect of the ligand indicates that once these two Class I variants lose structural NADP^+^, these enzymes became more unstable and lose activity, which is consistent with their causing the most severe disease phenotype.

All the mutants included in this work showed more hydrophobic regions exposed to solvent than the WT G6PD enzyme (Figure 6), and the Class I variants were no exception. Likewise, derivatization experiments showed that the Cys residues of all the mutants, including the Class I variants, are more accessible to DTNB than in the WT enzyme. This observation suggests that the mutant enzymes have much lower structural rigidity than the WT G6PD enzyme.

In this regard, computational analyses based on the 3-D (three-dimensional structure) showed that the residue arginine-227 (G6PD Mexico City variant) is located in a loop at the surface of the molecule and that substitution with a residue such as glutamine leads to steric hindrance, probably affecting the local conformation and thereby causing the disease phenotype. Furthermore, as in the Mexico City G6PD variant, the 3-D structural analysis of the Valladolid G6PD (R136C) showed that the residue arginine-136 is located in a β-sheet at the surface of the molecule. In both variants, the point mutations are distant from the dimer interface and the binding sites for structural NADP^+^, substrate and coenzyme.

**Figure 7:**
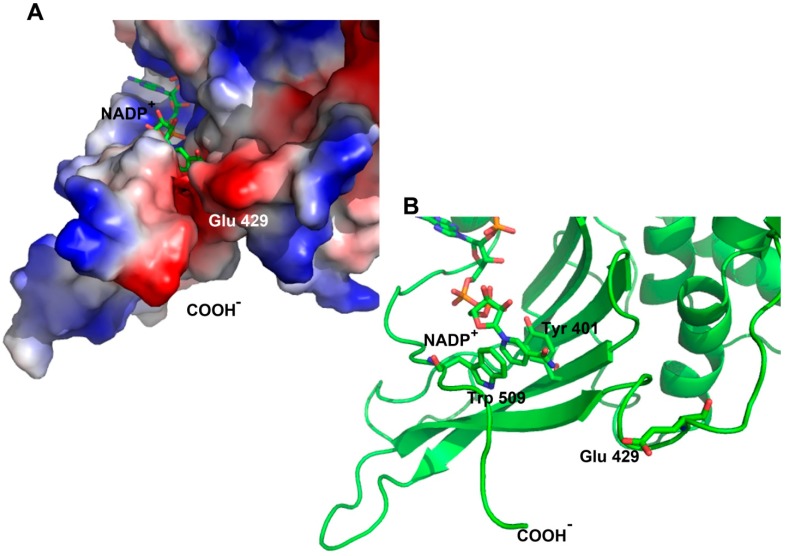
Schematic representation of human G6PD enzyme (PDB code 2BH9). (**A**) Surface electrostatic potential of the crystallographic structure of the G6PD; and (**B**) G6PD in ribbon format, shown are π–π interactions between the amino acid residues Try 509 and Tyr 401 with the pyramidal ring of NADP^+^. Modeled with PyMOL [30].

In the case of the Yucatan G6PD variant (K429E), we studied *in silico* mutation of the glutamic amino acid residue at position 429 (Glu 429) (Figure 7) over the crystallographic structure of G6PD (PDB code 2BH9), and the potential interactions of the mutated residue were analyzed. This residue is located on loop 21 and is highly exposed to solvent (approximately 70% accessibility). The mutation analysis shows that the OE2 of the side chain is oriented toward the *C*-terminus at a distance of 7 Å (Figure 7B). Additionally, electrostatic potential is generated as shown in Figure 7A: both the *C*-terminus and the mutated amino acid (Glu 429) exist in an environment of negative charge, which could promote repulsion due to the new amino acid with negative charge. The high mobility of the *C*-terminus and the repulsion of the new charge could increase instability, affecting interactions between structural NADP^+^ and the neighboring amino acids. For example, in neighboring amino acids, π–π interactions of Try 509 and Tyr 401 with the pyramidal ring of NADP^+^ (Figure 7B) could be disrupted, potentially affecting the affinity for structural NADP^+^ and consequently the global stability of G6PD. In this sense, it has been shown that the *C*-terminus is highly disordered, leading to lower occupancy of structural NADP^+^ [18]. Thus, charge repulsion could increase the disorder in the *C*-terminus and affect the binding of structural NADP^+^, which would lead to instability of the enzyme. It is interesting to note that although the point mutation K429E (Yucatan variant, Class I) has no direct interaction with the structural NADP^+^, it causes long-range conformational changes in the active site, decreasing the structural rigidity of the mutant enzyme, making it more unstable and diminishing its activity. This loss of activity is consistent with the severity of the disease phenotype it causes.

## 4. Experimental Section

### 4.1. Substrates and Reagents

Restriction enzymes, ligases and DNA molecular markers were purchased from New England Biolabs (Beverly, MA, USA). Agarose was purchased from Gibco BRL (Grand Island, NY, USA). DNA miniprep kits and gel extraction kits were purchased from Qiagen (Valencia, CA, USA). All the primers for both mutagenesis and DNA sequencing were purchased from the Biotechnology Institute of National Autonomous University of Mexico (UNAM; Mexico City, Mexico). 2',5'-ADP Sepharose 4B was purchased from Amersham Biosciences (Piscataway, NJ, USA). G6PD and NADP^+^ were obtained from Sigma Chemical Company (Poole, UK). Centricon Ultra centrifugal filter devices in units of YM-30 were used (Millipore, Billerica, MA, USA).

### 4.2. Construction of the E. coli Mutant Strain BL21(DE3)Δzwf::kan^r^

The strains and plasmids used in this work are listed in Table S1. *E. coli* strain BL21(DE3)Δ*zwf*::kan^r^ was constructed by P1*vir* transduction [15] of the Δ*zwf*::Kan^r^ allele from strain JW1841 [16] into *E. coli* BL21DE3 (Stratagene, Santa Clara, CA, USA). Lysates of phage were prepared as previously reported with minimal modifications [15,31,32]. Briefly, 1 × 10^9^ PFU (plaque forming units) of phage were combined with the donor bacterial strain 5 × 10^8^ CFU (colony forming units) supplemented with 10 mM calcium chloride and 5 mM magnesium sulfate and incubated for 5 min. The latter mixture was added with 3 mL of 0.8% LB top agar, overlaid onto fresh LB plates and incubated at 37 °C for 14 h. To allow the release of phage from the top agar, 7 mL of LB liquid medium were added and the top agar was scraped from the surface of the agar plate and transferred in a sterile tube to shake it slowly at room temperature for 1 h. To separate the phages of the bacteria and agar, the lysate was centrifuged at 17,000× *g* 5 min. The supernatant was transferred to a new sterile tube, and the remaining cells were lysed by the addition of 30 µL of chloroform, mixed 30 s in vortex and stored at 4 °C. Transductions were performed as previously recommended by Thomason *et al*. [15], with kanamycin as the selection marker carried by the donor strain. Typically, 100 μL of an overnight LB-grown culture (5 × 10^8^ CFU) was mixed with 30 μL of a phage lysate with a titer of 10^10^ to 10^11^ PFU/mL, supplemented with 10 mM calcium chloride and 5 mM magnesium sulfate and incubated at 30 °C without shaking for 30 min. After the incubation, 200 µL of 1 M Na-citrate pH 5.5 was added and centrifuged at 5000× *g* 5 min. The pellet was resuspended in 900 µL of LB medium supplemented with 100 mM Na-citrate pH 5.5 and incubated for 30 min at 37 °C with shaking. Next, the mixture was centrifuged at 12,000× *g* for 1 min, and the pellet was resuspended in 100 µL of LB medium and plated on LB agar medium supplemented with kanamycin (100 μg/mL). The transductant cells typically appeared after 16 h of incubation at 37 °C. The genotype of this mutant was subsequently confirmed by PCR mapping and phenotypic testing via enzymatic assay.

### 4.3. Construction of G6PD Mutants and Cloning into an Expression Plasmid

The recombinant plasmid pET-HisTEVP-*g6pd* containing the full human *g6pd* gene [17] was used as a template for mutagenesis. Two flanking primers (see Table 1) were designed for PCR amplification of the human *g6pd* gene. The primers introduced *Nde*I and *Bpu*11021 restriction sites (underlined) at the 5' and 3' ends, respectively. For the variants, the desired mutations in G6PDs Yucatan (K429E), Valladolid (R136C), Mexico City (R227Q) and Nashville (R393H) were generated by site-directed mutagenesis [22]. All PCR protocols were performed using a peltier-controlled Mastercycler gradient thermal cycler from Eppendorf (Hamburg, Germany). In the first-round PCR, the reaction mixture contained 0.2 µg of template pET-HisTEVP-*g6pd*, 200 ng of flanking *Nde*I primer, 200 ng of a mutagenic reverse primer containing the desired mutation (see Table 1), 200 nmol of dNTP mixture and 1 U Phusion High-fidelity DNA polymerase (Thermo Scientific, Hudson, NH, USA). The second-round PCR was performed using Bpu11021 reverse primer and the respective mutagenic forward primer (see Table 1) with the same conditions used in first-round PCR. The PCR conditions were as follows: 5 min at 98 °C for denaturation; 25 cycles of amplification (1 min at 98 °C, 1 min at 62 °C, 1 min at 72 °C) and 10 min at 72 °C for extension. A third round of PCR was performed to obtain the gene with the desired mutation using the same condition as first-round PCR with *Nde*I forward and *Bpu*11021 reverse primers, 200 ng of purified PCR1 and PCR2 products. All the PCR products for each mutant were analyzed by 1% agarose gel electrophoresis and amplicons were purified for each case with the QIAquick Gel Extraction Kit (Qiagen).

The PCR products of each mutant were then cloned in the pJET 1.2 vector (CloneJET PCR Cloning Kit; Thermo Scientific). The vectors containing the different mutations were named as follows: pJg6pd, pJgK429E, pJgR136C, pJgR227Q and pJgR393H. All vectors constructed were then inserted into competent *E. coli* BW25113 cells, which were grown at 37 °C overnight on an LB-agar plate containing 100 µg/mL ampicillin. Plasmid DNA of each mutant was isolated and full sequenced to ensure the fidelity of the sequence. Overlapping sequences were obtained for each mutant using internal forward and reverse sequencing primers (Table 1). The pJET 1.2 vector containing the verified sequence for each mutant human *g6pd* gene was digested with *Nde*I and *Bpu*11021 and sub-cloned into the pET-3a plasmid (Novagen, Madison, WI, USA). We named the plasmids pETg6pd, pETgK429E, pETgR136C, pETgR227Q and pETgR393H. All constructs with the desired mutations were transformed into competent *E. coli* BL21(DE3)Δ*zwf*::kan^r^ cells, which were grown at 37 °C overnight.

### 4.4. Expression and Purification of Recombinant G6PD Enzymes

First, the optimal expression conditions were standardized in LB culture medium using three IPTG concentrations (0.1, 0.5 and 1 mM, respectively) and three different temperatures (15, 25 and 37 °C), which were tested during 18-h expression time courses. Samples were taken at different time points (2, 6, 12 and 18 h), and the specific activity was measured. At the indicated times, the cells were concentrated by centrifugation, resuspended in lysis buffer (0.1 M Tris-HCl, pH 7.6, 3 mM MgCl_2_, 0.5 mM PMSF and 0.1% β-mercaptoethanol) and disrupted by sonication. The cell extract was centrifuged at 23,000× *g* for 30 min at 4 °C, and aliquots from the supernatant were used to quantify protein concentration and to calculate specific G6PD activity. The expression of the WT, Yucatan, Valladolid, Mexico City and Nashville G6PD enzymes was conducted at the best expression temperature, IPTG concentration and expression time for each case. The WT enzyme and G6PD mutants were grown at 37 °C for 5 h until O.D._600 nm_ = 1.0. The cells were centrifuged, resuspended in lysis buffer and disrupted by sonication. The crude extract was centrifuged at 23,000× *g* for 30 min, and the supernatant was used for the purification of the respective enzymes. The supernatant was fractionated with ammonium sulfate to 25%, incubated at 4 °C for 2 h and centrifuged at 23,000× *g* for 30 min at 4 °C. The supernatant was precipitated with 50% ammonium sulfate, incubated at 4 °C for two hours and centrifuged as indicated. The pellets were dissolved in 10 mM phosphate buffer, 0.15 M NaCl, pH 7.4 (binding buffer) and applied to a 2',5'-ADP Sepharose 4B affinity column (GE Healthcare, Piscataway, NJ, USA) pre-equilibrated with the binding buffer. The column was washed with 50 mM potassium phosphate buffer containing 1 mM EDTA, 1 mM DTT, and 80 mM KCl at pH 7.35. The wash was continued until the absorbance decreased to zero at 280 nm. Finally, the G6PD enzymes were eluted with 80 mM potassium phosphate buffer containing 80 mM KCl, 1 mM EDTA, plus 100 µM NADP^+^ at pH 7.85. Fractions showing G6PD activity were pooled and concentrated using Centricon YM-50 filtration tubes (Millipore). Next, the protein samples were dialyzed against 50 mM potassium phosphate buffer, pH 7.35 and then applied to a Q-sepharose-4B column (Sigma–Aldrich, St. Louis, MO, USA) pre-equilibrated with 50 mM potassium phosphate buffer, pH 7.35. A wash with the same buffer was performed until the effluent absorbance decreased to zero at 280 nm. The G6PD enzymes were eluted using a linear gradient from 0 to 0.35 M NaCl in the same buffer. Fractions showing G6PD activity were pooled and concentrated in Amicon YM-30 tubes (Millipore Corp., Bedford, MA, USA). The NaCl was removed from the sample by 3 successive steps of tenfold dilution in 0.025 M phosphate, pH 7.4. The purification steps were analyzed by 10% SDS-PAGE gels stained with 0.05% Coomassie brilliant blue R-250 (Sigma–Aldrich). Protein concentration was quantified as previously described by Lowry *et al*. [33] using bovine serum albumin as the standard. The proteins were then preserved in ultra-pure glycerol (Sigma–Aldrich) at −70 °C. The purity of the recombinant enzymes was verified by SDS-PAGE.

### 4.5. Kinetic Characterization of WT G6PD and Variants

The enzymatic activity of WT G6PD and the Yucatan, Valladolid, Mexico City and Nashville variants was assayed spectrophotometrically at 25 °C by monitoring the reduction of NADP^+^ at 340 nm [34]. Standard reaction mixtures contained 0.1 M Tris-HCl buffer, pH 8.0, 0.01 M MgCl_2_, 0.2 mM NADP^+^ and 1 mM glucose-6-phosphate (G6P). One unit (U) of G6PD activity is the amount of enzyme required to produce 1 µmol of NADPH per minute per mg of protein. The initial velocity data were obtained by varying one substrate in a concentration range from 2.5 to 200 µM while the second substrate was fixed at saturating concentration. *V*_max_ and *K*_m_ were calculated by fitting the initial velocity data to the Michaelis-Menten equation (*v_i_* = *V*_max_∙[S]/*K*_m_ + [S]) by non-linear regression calculations. The value of *k*_cat_ was calculated from *V*_max_ considering a molecular mass of 59 kDa for the monomer.

### 4.6. Analysis of Secondary Structure, Thermal Stability, Thermoinactivation and Hydrophobic Surface Exposure

The structural parameters of WT G6PD and the Yucatan, Valladolid, Mexico City and Nashville variants were evaluated spectroscopically by CD. Far-UV CD spectra were recorded at 25 °C in a Jasco J-810 spectropolarimeter (Jasco Inc., Easton, MD, USA) equipped with a peltier-thermostated cell holder. Spectral scans ranging from 200 to 260 nm at 1 nm intervals were performed in a quartz cuvette with a path length of 0.1 cm. The assays were conducted with a protein concentration of 0.8 mg/mL. Assays with ANS were conducted in 25 mM phosphate buffer, pH 7.4 at 25 °C in a Perkin-Elmer LS 55 spectrofluorometer (Perkin Elmer, Wellesley, MA, USA) using an excitation wavelength of 395 nm and recording emission spectra from 400 to 600 nm with excitation and emission slits of 10 and 10 nm, respectively. The final concentrations of ANS and the G6PD were 165 μM and 400 μg/mL, respectively. Background fluorescence from the buffer was subtracted from the sample read [22]. Quantification of cysteine content was performed using Ellman’s reagent (Sigma–Aldrich) [23]. Briefly, reduced G6PD enzyme (200 μg/mL) was added to a cuvette with TE buffer, 25°C; after the addition of DTNB (1 mM), Cys quantification was conducted by recording absorbance at 412 nm. Finally, 5% SDS was added to completely denature the enzyme.

The thermal stability and unfolding of WT G6PD and the variants at 0.8 mg/mL in 25 mM phosphate buffer was measured as the change in the CD signal at 222 nm during temperature scans ranging from 25 to 70 °C at a rate of 1 °C per 2.5 min. The average temperature at which 50% of the protein is folded and 50% is unfolded is expressed as the melting temperature (*T*_m_) and was calculated as previously reported [21,22]. For thermoinactivation assays, the concentrated enzymes were diluted at a final protein concentration of 200 µg/mL in 50 mM potassium phosphate buffer pH 7.35 containing 2 mM MgCl_2_ and different concentrations of NADP^+^ (0, 5, 10, 100 and 500 µM). These samples were incubated for 20 min at a temperature between 37 and 65 °C and then cooled at 4 °C in a Peltier-controlled Mastercycler Gradient Thermal Cycler (Eppendorf) for 5 min. Residual activity was determined by triplicate experiments at 25 °C for WT G6PD and the four variants and expressed as a percentage of the activity of the same enzyme incubated at 25 °C.

## 5. Conclusions

In summary, the results of this study show that the deficiency caused by the point mutations in the Yucatan (K429E) and Nashville (R393H) severe Class I G6PD mutants and the Valladolid (R136C) and Mexico City (R227Q) mild Class II and III G6PD mutants affect catalytic efficiency and stability, and the enzymes exhibit a less compact 3-D structure than WT G6PD. There is no clinical data about these four G6PD deficiency mutants, and there is only limited information about G6PD Yucatan (K429E). Both G6PDs Yucatan and Nashville belong to Class I and show less than 5% G6PD activity in red blood cells. Both mutants exhibited a loss of catalytic efficiency, dramatically decreased stability and a requirement for high concentrations of NADP^+^ to compensate for these defects, correlating with the disease of chronic non-spherocytic hemolytic anemia. Although the G6PD variants Valladolid (R136C) and Mexico City (R227Q) are mild Class II and Class III mutations, they were affected in their catalytic efficiency and showed slight changes in their compact 3-D structure, consistent with the phenotype of the disease as dependent on environmental challenge or on the individual genetic background. Furthermore, because mature erythrocytes lack a nucleus and cannot synthesize proteins, the protein instability of the mutant enzymes is clearly compatible with the predominant symptoms: In non-nucleate mature erythrocytes, enzymes are required to survive several months or longer in circulation.

## Supplementary Materials

Supplementary materials can be found at http://www.mdpi.com/1422-0067/15/11/21179/s1.
